# Differentiating the potential of continuum beliefs to reduce the public stigma of schizophrenia, depression, and alcohol dependence: associations with illness recognition and treatment recommendations

**DOI:** 10.3389/fpsyt.2026.1869356

**Published:** 2026-09-07

**Authors:** Sven Speerforck, Anya Leonhard, Matthias C. Angermeyer, Georg Schomerus

**Affiliations:** 1 Department of Psychiatry and Psychotherapy, University Hospital Leipzig, Leipzig, Germany; 2 Institut für Sozialpsychiatrie Mecklenburg-Vorpommern e.V., Rostock, Germany; 3 Department of Public Health, Clinical and Molecular Psychiatry, University of Cagliari, Cagliari, Italy; 4 Center for Public Mental Health, Gösing am Wagram, Austria

**Keywords:** continuum beliefs, illness recognition, mental health literacy, public stigma, stigma

## Abstract

**Purpose:**

Promoting continuum beliefs has been discussed as a strategy for stigma reduction. The current study examines associations of continuum beliefs with stigma aspects (stereotypes, emotions, and social distance) and mental health literacy (illness recognition and treatment recommendations).

**Methods:**

In 2011, a sample of the German population (*N* = 3,642) completed a vignette-based interview on attitudes regarding schizophrenia, depression, or alcohol dependence. Logistic regression analyses were performed and predicted probabilities were used to calculate prevalence estimates and depict direct effect sizes.

**Results:**

Increased continuum beliefs were strongly related to a *decreased* likelihood of social distance and endorsement of some negative stereotypes (e.g., being alien for schizophrenia) and an *increased* likelihood for endorsement of other negative stereotypes (e.g., lack of willpower for schizophrenia and depression) and emotions (e.g., anger for schizophrenia). Effect sizes indicated a greater reduction in negative stereotypes and desired social distance than increases in negative stereotypes or emotions. Furthermore, continuum beliefs were associated with a *decreased* probability of illness recognition for schizophrenia and recommending treatment for schizophrenia and depression, but an *increased* likelihood for illness recognition and recommending treatment for alcohol dependence.

**Conclusion:**

Potentially harmful associations of continuum beliefs were found for schizophrenia and depression and should be considered by future studies and intervention designs. These potentially harmful associations are likely outweighed by helpful associations based on prevalence estimates and effect sizes. Associations between continuum beliefs and mental health literacy seem to be illness-dependent. Because of the sociocultural context and the time gap between the study and publication, representativeness for current public attitudes is limited.

## 1 Introduction

People with mental illnesses are subjected not only to symptoms, but also to public attitudes and opinions regarding their illness. These attitudes include differing levels of knowledge about mental illnesses and treatment (“mental health literacy”) (1, 2), emotional reactions such as fear, or stigmatizing beliefs and wishes, such as desired social distance or negative stereotypes (3). The stigma of mental illness has been described as pervasive, with a significant impact to the quality of life of the affected people (4). Both stigma and mental health literacy vary between different types of mental illnesses (3, 5, 6). One approach for stigma reduction is promoting certain concepts of mental illness, such as continuum beliefs, which conceptualize mental illness and health as existing on a spectrum as opposed to being distinct categories (7, 8). Previous studies have found a rather consistent association between continuum beliefs, lower desired social distance, and agreement with negative stereotypes as well as higher prosocial reactions (8).

As continuum beliefs highlight perceived similarities between people who are affected and those who are unaffected by mental illness (cf. 9), they may impact whether experiences of mental illness (i.e., symptoms) are interpreted as pathological. Illness recognition is an important aspect of mental health literacy and has been associated with a higher likelihood of recommending treatment (10). Fear as an emotional reaction, which has been negatively associated with continuum beliefs (8), has also been a predictor of recommending treatment for alcohol dependence, depression, and schizophrenia (11). Similarly, higher biogenetic causal beliefs, which are often described as antithetical to continuum beliefs (cf. 12), have been associated with an increased likelihood of recommending treatment in general for schizophrenia as well as medication for both depression and alcohol use disorder (11). There thus seems to be a tension between the established positive associations with continuum beliefs and the possibility that promoting continuum beliefs may inadvertently hinder illness recognition and recommending appropriate help-seeking strategies.

Previous studies have not yet examined specific associations between continuum beliefs and treatment recommendations of the public. Studies have shown tentative positive results for increasing recognition of risky drinking through continuum concepts, but have not examined whether increased problem recognition relates to positive attitudes toward treatment utilization for oneself or others (13, 14). Interestingly, despite the consistent correlation between higher continuum beliefs and lower fear in analytical studies, one interventional study found that promoting continuum beliefs increased fear for schizophrenia, possibly through the notion that schizophrenia may become “too close for comfort” (15).

It thus seems particularly important to establish the strength of the associations between continuum beliefs and individual attitudes that are positive (e.g., lower social distance and lower agreement with harmful stereotypes) or negative (e.g., lower recommendations for treatment) to estimate the relevance of continuum beliefs for each, while considering differing prevalences of both continuum beliefs and specific stigmatizing attitudes for different disorders.

In this study, we re-analyzed data from a large population survey conducted in Germany in 2011 to examine how a belief in a continuum of symptoms is related to the recognition as mental illness, treatment and help-seeking recommendations, stereotypes, emotions, and desire for social distance. Additionally, we aimed to address which attitudes are more strongly related to continuum beliefs through utilizing predicted probabilities to depict direct effect sizes. We aimed to address the following questions:

- Is the belief in a continuum of symptoms related to a lower recognition as mental illness or less recommendations of professional help?
- How are beliefs in a continuum of symptoms related to stereotypes, emotions, and desire for social distance?
- Do these associations differ between mental illnesses like schizophrenia, depression, and alcohol dependence?
- What are the effect sizes of continuum beliefs considering the absolute prevalence estimates of the investigated variables?

## 2 Methods

### 2.1 Participants

A population survey of German-speaking adults (>18 years) living in private households in Germany was conducted in two waves during March and April 2011 and November and December 2011. The sample was drawn using a random sampling procedure with three stages: (1) electoral wards, (2) households, and (3) individuals within the target households. Target households within the sample points were determined according to the random route procedure (16); target persons within households were selected by random digits. Informed consent was considered to have been given when individuals agreed to complete the interview. Fieldwork was done by USUMA (Berlin), a company specialized in market and social research. Protection of privacy prohibited the collection of any information on non-respondents. The study was approved by the institutional review board of the responsible university. Altogether, 3,642 persons completed the interview, reflecting a response rate of 64.0%. Although containing slightly more women and less well-educated and single respondents, our sample can be considered largely representative of the German population (**Supplementary Table 1**). Data are available from the corresponding author upon request.

### 2.2 Interviews and case vignettes

Personal, fully structured interviews were conducted face to face. The interview started with presenting an unlabeled case vignette of a person with schizophrenia, major depression, or alcohol dependence. The gender of the person described in the vignette varied at random. The wording of the vignettes was constructed to be consistent with the diagnostic criteria of the respective disorders in *Diagnostic and Statistical Manual of Mental Disorders, 4th Edition* (*DSM-IV*) and had undergone validation by five blinded and board-certified experts in psychopathology (17). The exact wording of the used vignettes was published earlier (18).

### 2.3 Measures

#### 2.3.1 Continuum beliefs

As reported earlier (19), we asked respondents to indicate their agreement with the following statement: “Basically, we are all sometimes like this person. It’s just a question of how pronounced this state is”. Answers had to be given on a five-point Likert-scale, with “1” indicating strong agreement and “5” indicating strong disagreement with the statement. We reversed the scores for our analysis and used it as a continuous variable with higher scores indicating stronger agreement with the respective statement.

#### 2.3.2 Illness recognition

Following the presentation of the unlabeled case vignette, we asked respondents whether they considered the person described as having a mental illness in a medical sense. Answers were coded as “don’t know”, “no”, and “yes”.

#### 2.3.3 Help-seeking and treatment recommendations

Participants were presented with a list of help-seeking and treatment recommendations that was designed to capture both lay and professional recommendations and was similarly used in earlier studies (20). In this paper, we focus on the recommendation to see a psychiatrist and the two treatment options “psychotherapy” and “psychotropic medication”. The strength of each recommendation was rated on a five-point Likert scale, with “1” = recommend urgently, “5” = strongly advise against, and “6” = don’t know for undecided opinions. To facilitate multinomial logistic regression analysis, we reduced the six dimensions of the variable to four new dimensions: 1, 2 = recommendation; 3 = neutral; 4, 5 = don’t recommend; 6 = don’t know.

#### 2.3.4 Stereotypes

The respondents were presented with a list of personal attributes including “dangerous”, “unpredictable”, “being alien”, and “lack of will power”. They were asked to indicate, with the help of a five-point Likert scale ranging from “1” = applies completely to “5” = does not apply at all, to what extent these attributes apply to the person depicted in the vignette. For our analysis, we reduced the dimensions accordingly: 1, 2 = apply; 3 = neutral; 4, 5 = does not apply.

#### 2.3.5 Emotional reactions

Following the stigma concept of Link and Phelan (21) and Link et al. (22), negative emotional reactions are an important consequence of negative stereotypes, leading to separation, discrimination, and status loss. To investigate emotional reactions toward the person with depicted symptoms, we used the “Emotional Reactions to the Mentally Ill Scale (ERMIS)” (23). Respondents were presented with a scale consisting of 10 items describing possible emotional reactions, asking them to indicate how they would react to the person described in the vignette. Answers were given on five-point Likert scales anchored with “1” = applies completely and 5 = “does not apply at all”. In this study, we used the items “feel uncomfortable”, “person is provoking fear”, “react angrily”, “feeling annoyed”, and “feel sympathy”. For our analysis, we reduced the dimensions as described above.

#### 2.3.6 Desired social distance

*The desire for social distance* is a widely used measure of individual discrimination and thus represents the final stage of the stigma process (21, 22). With the help of a five-point Likert scale, respondents were asked how willing they would be to introduce the depicted person to a friend (“1” = definitely; “5” = definitely not). For our analysis, we reduced the dimensions as described above.

### 2.4 Statistical analysis

Fourteen different multinomial logistic regression analyses were performed with illness recognition, recommendations, stereotypes, emotional reactions, and the willingness to introduce the person to a friend as dependent variables, with a hybrid of ordinal (1–3) and nominal (don’t know) responses. Predictors comprised belief in a continuum of symptoms and vignette condition as well as age, gender, and education using the unweighted survey data. To estimate the probabilities for the three different illnesses, an interaction term with the vignette condition was included and the respective standard deviation of the continuum belief was calculated for each vignette condition separately. Instead of relative risk ratios, we estimated probabilities for the categories of the dependent variables and related probability differences, which straightforwardly can be interpreted as prevalence estimates and related effect sizes. The effect of the continuum belief on the probability of a given category is calculated as the probability difference (discrete probability change) for a change of one standard deviation of the continuum belief as predictor. The figures thus show the predicted probabilities for the “agree”, “recommend”, “apply”, and “introduce” category holding all predictors at their means, and the predicted probabilities with an increase of the continuum belief by one standard deviation and the related probability change. **Supplementary Tables 2**–**4** display results of the full models comprising all other possible response categories and related confidence intervals. Because of rounding, the predicted probability change was not always equal to the difference between predicted probabilities. Probabilities and their differences were calculated for each vignette condition using the module “mlincom” from the SPost13 Suite programmed for Stata 13 (24). Computations were carried out using Stata 13.1 SE (25). The code used for analysis is available upon request.

The prevalence estimates of the investigated variables [illness recognition in Schomerus et al. (19)] have been published previously (26).

## 3 Results

**Figure 1:**
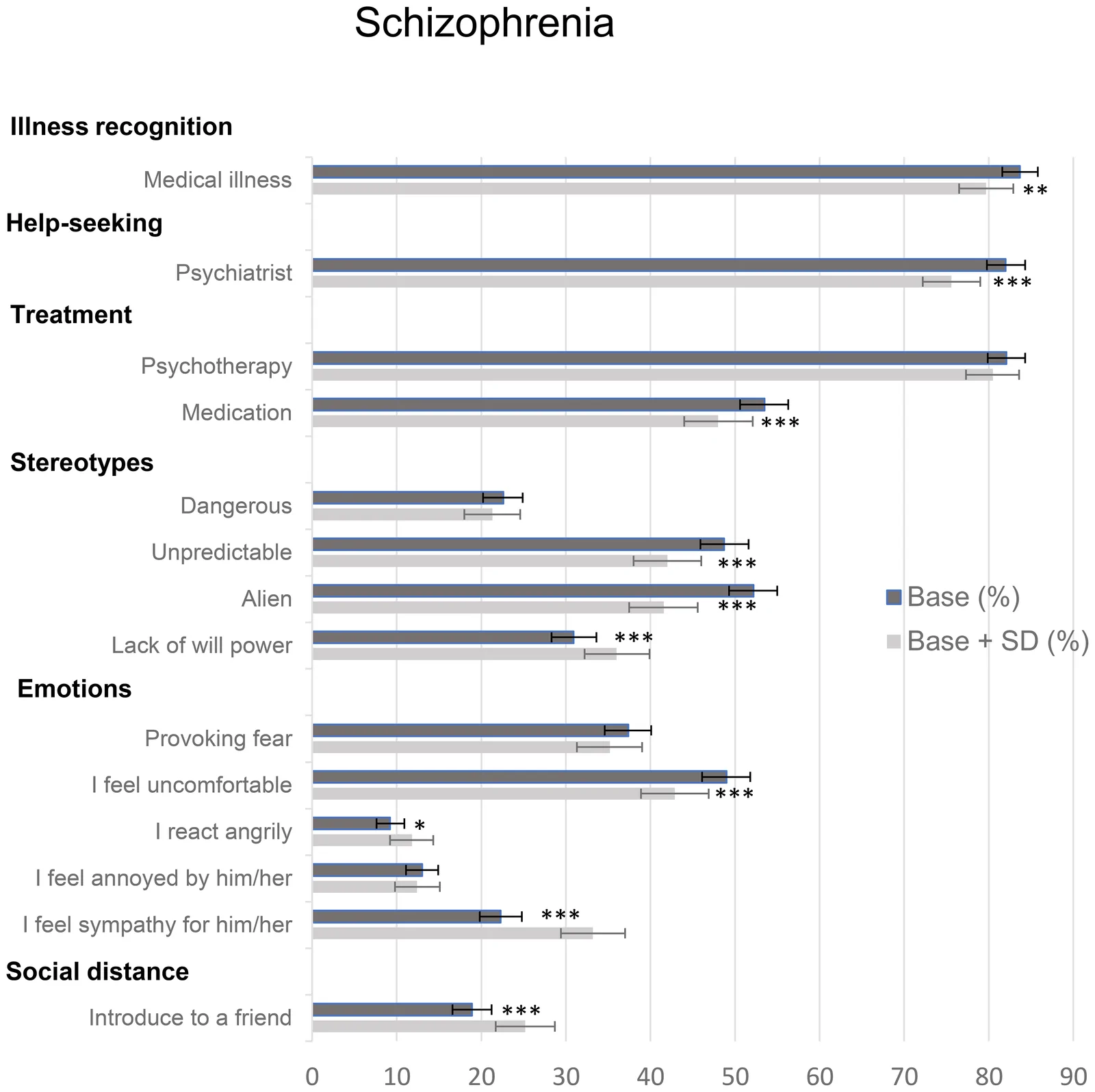
Recognition of medical illness, recommendations for help-seeking and treatment, emotional reactions, stereotypes, and desire for social distance after presentation of unlabeled case vignettes describing symptoms of schizophrenia meeting *DSM-V* criteria. Multinomial regression analyses, controlled for socio-demographic variables, predicted probabilities of the affirmative category with continuum belief at its mean [Base (%)] and the increase of the continuum belief variable by one standard deviation [Base + SD (%)] and its 95% confidence intervals. The estimated probabilities for the dependent variables and the related probability differences can be interpreted as prevalence estimates and effect sizes. Total *N* in full models was 3,551–3,560 after missing values were excluded. *p < 0.05, **p < 0.01, ***p < 0.001.

**Figure 2:**
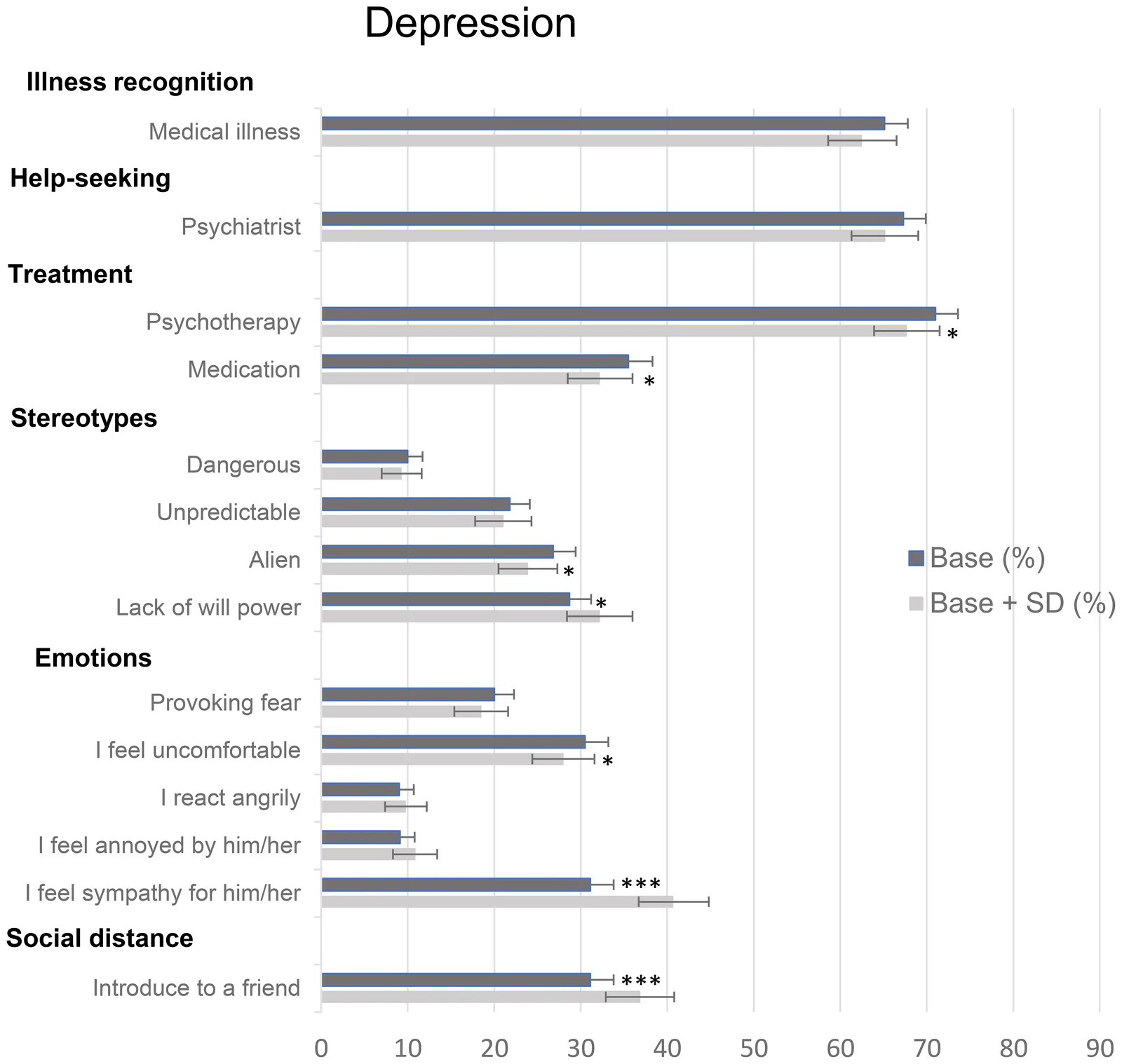
Recognition of medical illness, recommendations for help-seeking and treatment, emotional reactions, stereotypes, and desire for social distance after presentation of unlabeled case vignettes describing symptoms of depression meeting *DSM-V* criteria. Multinomial regression analyses, controlled for socio-demographic variables, predicted probabilities of the affirmative category with continuum belief at its mean [Base (%)] and the increase of the continuum belief variable by one standard deviation [Base + SD (%)] and its 95% confidence intervals. The estimated probabilities for the dependent variables and the related probability differences can be interpreted as prevalence estimates and effect sizes. Total *N* in full models was 3,551–3,560 after missing values were excluded. *p < 0.05, **p < 0.01, ***p < 0.001.

**Figure 3:**
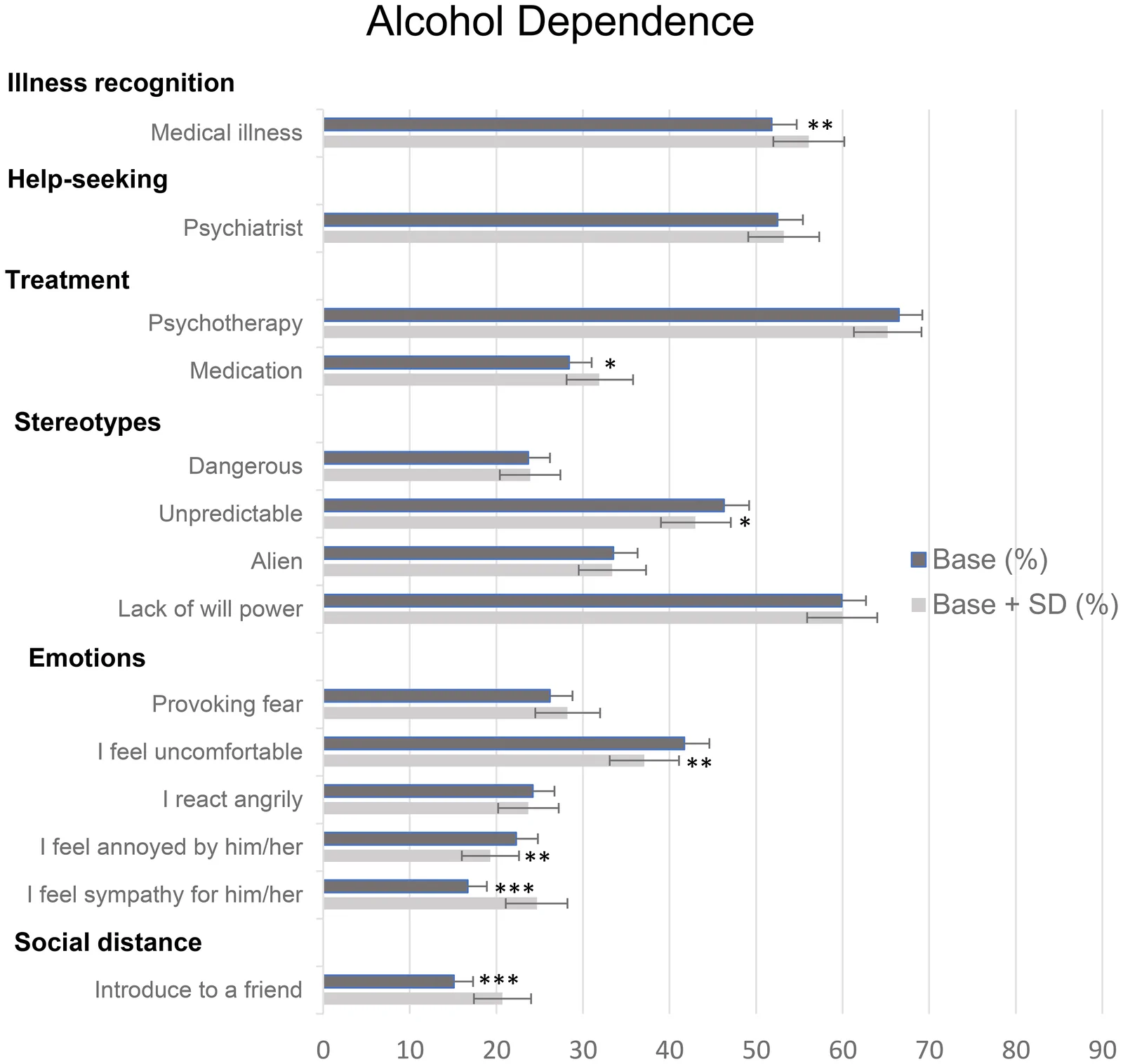
Recognition of medical illness, recommendations for help-seeking and treatment, emotional reactions, stereotypes, and desire for social distance after presentation of unlabeled case vignettes describing symptoms of alcohol dependence meeting *DSM-V* criteria. Multinomial regression analyses, controlled for socio-demographic variables, predicted probabilities of the affirmative category with continuum belief at its mean [Base (%)] and the increase of the continuum belief variable by one standard deviation [Base + SD (%)] and its 95% confidence intervals. The estimated probabilities for the dependent variables and the related probability differences can be interpreted as prevalence estimates and effect sizes. Total *N* in full models was 3,551–3,560 after missing values were excluded. *p < 0.05, **p < 0.01, ***p < 0.001.

**Figures 1**–**3** show the results of the multinomial regression analysis relating the probability of the affirmative category of different dependent variables to the belief in a continuum of symptoms. The full models including all categories (neutral, rejection, and “don’t know”) are presented in **Supplementary Tables 2**–**4**. The probability differences were drawn from the models adjusted for the named covariates in the full model. Results are reported as the predicted change in attitudes associated with an increase in continuum beliefs of one standard deviation in percent.

### 3.1 Schizophrenia

Recognition as medical illness and recommendations to see a psychiatrist and to get psychotherapy or psychotropic medication were highest when compared to depression or alcohol dependence. The belief in a continuum of symptoms predicted a lower probability of perception as medical illness (−4%) and of recommending psychotropic drugs (−6.4%) or a psychiatrist (−5.4%). Regarding stereotypes, being unpredictable and alien were the most often associated personal attributes. The probability of both was notably reduced by a higher belief in a continuum of symptoms (−6.7% and −10.6%, respectively), whereas the likelihood of assuming a “lack of will power” was increased by a higher belief in a continuum of symptoms (+5.1%). The belief in a continuum of symptoms predicted a lower probability of the most frequently mentioned emotion of “feeling uncomfortable” (−6%) and a much higher probability of “feeling sympathy” (+10.9%). Although the likelihood of the least frequent emotion “react angrily” was slightly increased by a higher continuum belief (+2.5%), it predicted a higher probability of introducing the depicted person to a friend (+6.3%). The probability of the perception as dangerous or to provoke fear was not associated with the continuum belief.

### 3.2 Depression

The likelihood of recommending psychotherapy or psychotropic medication for a person with major depression was slightly reduced by a higher continuum belief (−3.2% and −3.3%, respectively). A probability change of perceiving the depicted person as being alien (−2.9%) or with a lack of will power (+3.5%) was associated with a higher belief in a continuum of symptoms. In comparison with schizophrenia, the estimated probabilities were generally higher for “feeling sympathy” or “introduce to a friend” and lower for “feeling uncomfortable”. Whereas the estimated effect size of the continuum belief on “feeling uncomfortable” was statistically significant but low (−2.5%), the predicted probability changes of “feeling sympathy” (+9.6%) and “introduce to a friend” (+5.8%) were comparable to those predicted for a person with schizophrenia.

### 3.3 Alcohol dependence

The view that the depicted symptoms of alcohol dependence are representative of a medical illness and recommendations for professional help were generally lower, when compared to schizophrenia or depression. Only regarding alcohol dependence did a higher belief in a continuum of symptoms predict a higher probability of recognizing the person as having a medical illness (+4.3%) or of recommending psychotropic medication (+3.5%). Whereas there was no predicted probability change regarding the most often endorsed stereotype of “lack of will power”, a higher belief in a continuum of these symptoms reduced the probability of the stereotype of being unpredictable (−3.2%). With regard to emotions and desire for social distance, a higher belief in a continuum of symptoms was related to lower probabilities for feeling uncomfortable (−4.7%) or annoyed (−3.0%) and to higher probabilities for feeling sympathy (+8.0%) or for introducing the depicted person to a friend (+5.6%).

The explanation of variance (Pseudo *R*²) of our models was generally low (2.3%–5.8%, details can be found in the **Supplementary Materials**).

## 4 Discussion

Re-analyzing the data, we aimed to investigate the relationship of continuum beliefs with mental health literacy variables, comparing it with established stigma variables by using effect sizes. Study participants who endorsed higher continuum beliefs were less likely to recognize schizophrenia as an illness and less likely to recommend different treatment modalities for schizophrenia and depression. In contrast, study participants with higher continuum beliefs showed a higher likelihood of both illness recognition and recommending medication for alcohol dependence. For schizophrenia, results could reflect that perceptions of psychosis as a spectrum potentially normalize symptoms (cf. 27; cf. 28) when the overall percentage of illness recognition is high. For alcohol dependence, increased continuum beliefs could be related to a broader illness concept through undermining the dichotomization of healthy and unhealthy alcohol use. Taking into account alcohol use disorder as a heavily stigmatized condition (29) and that only 52% of respondents considered the depicted person as having a mental illness, continuum beliefs may enable recognition of drinking behavior as potentially problematic and thus increase the perception that the affected person may need medical treatment. Previous studies have similarly shown that various continuum narratives may boost self-recognition of risky drinking (13, 14). Overall, the relationship between continuum beliefs and illness recognition seems to be complex and illness-dependent.

Regarding different stereotypes, an increase in continuum beliefs showed mixed results for stereotype agreement. Continuum beliefs predicted a lower probability of negative stereotype agreement such as being “alien” for both schizophrenia and depression and “unpredictable” for schizophrenia and alcohol dependence, but a higher likelihood of perceiving a “lack of will power” for schizophrenia and depression. Overall, results are consistent with prior literature, which have indicated that continuum beliefs are significantly negatively associated with perceptions of distance (8).

A positive association between continuum beliefs and a higher likelihood of feeling sympathy and introducing the person in the vignette to a friend was found for all three vignette conditions. Previous literature has similarly indicated that higher prosocial reactions and lower desired social distance are fairly consistently associated with continuum beliefs (8). Other results include that higher continuum beliefs predicted a lower probability of being uncomfortable for all illnesses, a lower probability of being annoyed for alcohol dependence, and a higher probability of anger for schizophrenia, although the prevalence of endorsing a feeling of anger was generally low. A positive association between continuum beliefs and anger has been previously described by Angermeyer and colleagues (30), who speculate that when an affected person’s behavior is not attributed to an illness, it may provoke more anger. This may also explain the association with perceiving a “lack of will power” for schizophrenia and depression. While this finding may seem contrary to the emphasis on similarities that go hand in hand with continuum beliefs, endorsing a “lack of will power” or reacting with anger may be attempts to re-establish distance to the affected person in the vignette. As the thought of developing a severe mental illness is generally distressing, a person with high continuum beliefs may believe that affected people simply have lower will power in order to explain why they themselves are not vulnerable to developing this type of mental illness. Similarly, people with high continuum beliefs may react with more anger due to a decreased understanding of a person’s behavior as part of an illness entity, thus shifting more personal responsibility onto the affected person. The study found no relationship between continuum beliefs and fear. Similarly, the likelihood of endorsing the “dangerousness” stereotype was not associated with an increase in continuum beliefs. Findings indicate that perceptions of dangerousness are not strongly associated with perceptions of similarity, as there was a decreased probability of agreeing with the “alien” stereotype with higher continuum beliefs. This may be because people see negative aspects of themselves in vignette descriptions or assume the worst of themselves. Thibodeau and colleagues have described a continuum intervention for schizophrenia stigma that increased participants’ self-reported fear, possibly due to feeling too close to the person featured in the intervention (15).

This study is the first to directly compare potential beneficial or harmful relations with an increase of continuum beliefs through using predicted probabilities as a marker for effect size. For the schizophrenia vignette, the greatest absolute and relative changes in effect sizes were mostly found for positive changes such as lower endorsement of the “alien” stereotype (−10.6%), likelihood of introducing the person to a friend (+6.3%), and “feeling sympathy” (+10.9%) compared to potential negative consequences [e.g., reacting angrily (+2.5%) and perceiving a lack of will power (+5.1%)]. The probability change for illness recognition, recommending drugs, and recommending psychiatric treatment was between −4% (illness recognition) and −6.4% (recommending psychotropic drugs). Considering the high base level of illness recognition and treatment recommendations, these changes appear relatively small in size. This indicates that the relationship between continuum beliefs and measures of reduced stigma (specifically increased sympathy and decreased social distance) seems potentially more relevant than the relationship between continuum beliefs and negative stereotypes and emotions (e.g., “lack of will power” and “I react angrily”) as well as treatment recommendations and illness recognition.

The probability changes for depression were similarly high for “feeling sympathy” (+9.6%) and “introduce to a friend” (+5.8%), while probability changes for the negative stereotype “lack of will power” (+3.5%) and lower likelihood of recommending psychotherapy (−3.2%) or medication (−3.3%) were lower. Despite these associations between continuum beliefs and negative attitudes, results indicate that there are stronger relationships between continuum beliefs and positive attitudes.

For alcohol dependence, increased continuum beliefs were consistently associated with measures of reduced stigma (e.g., lower likelihood of endorsing the stereotype of unpredictability and feeling annoyed, higher probability for feeling sympathy and introducing the affected person to a friend) and, interestingly, a higher likelihood of illness recognition and recommending medication.

### 4.1 Limitations

Our results should be interpreted in the light of the limitations of our study. First, to investigate effects on illness recognition, we used unlabeled case vignettes, while reactions to labeled vignettes or persons with the described mental illnesses might differ. Second, interviews were conducted face to face, which carries a risk for social desirability and agreement bias. Third, we conducted an observational study yielding for probability changes. No experimental design was included, and it is important to stress that no statements regarding causality can be made. Fourth, since the explanatory power of our models was low, there are probably other factors associated with the dependent variables that were not examined in our study. Fifth, the use of a single item measuring continuum beliefs, though well-established, may have diminished our ability to assess associations between different concepts of symptom continuity (31). Our sample had a slightly higher percentage of women and a lower percentage of well-educated and single respondents when compared to the general population of Germany. Women and higher-educated people have been shown to exhibit less stigmatizing attitudes in other sociocultural contexts (32, 33). Gender and education were thus included as covariates in our analysis. Finally, our study was conducted in Germany in 2011. Results may differ in other sociocultural contexts or at the time of publication. While there have been a multitude of local anti-stigma campaigns as well as significant events such as the 2015 suicide by aircraft by a Germanwings pilot, these are unlikely to have caused significant shifts in public opinion (3, 34), and stigma remains a highly relevant topic for all three disorders discussed in this study. Between 1990 and 2020, there was only a slight improvement in stigma toward depression, while stigma toward schizophrenia increased. Between 2011 and 2020, continuum beliefs regarding schizophrenia decreased while those regarding depression increased (35). The correct identification of both schizophrenia and depression in studies of the German population increased between 1990 and 2020, indicating improved mental health literacy, though the use of medical language increased only slightly for schizophrenia (+2.1%) between 2011 and 2020 compared to previous increases (+11.7% between 2001 and 2011) (6). Regarding alcohol dependence, longitudinal studies performed in other sociocultural contexts showed relatively stagnant or even regressive opinions in a similar time frame (36). The association between continuum beliefs and reduced likelihood of problem recognition for schizophrenia could be less relevant for the current population. Future studies should aim to compare associations between attitudes across different points in time, not just prevalences of certain beliefs.

On the other hand, our study has several strengths. First, the predicted probability changes can be interpreted as effect sizes and are based on a large population survey. Second, we provide the largest interview-based study investigating the associations of continuum beliefs with different attitudes and beliefs. Third, we investigated all three illnesses separately in unlabeled case vignettes, avoiding possible bias due to the use of medical vocabulary.

### 4.2 Implications

The findings of our cross-sectional study highlight that a potential decrease in illness recognition and recommendations for psychiatric treatment, specifically for schizophrenia, should be considered as drawbacks of interventions that promote continuum beliefs. Future studies should establish whether continuum interventions affect illness recognition and treatment recommendations and if such an effect could be avoided by including specific information about diagnostic categorization of symptoms and treatment options. Additionally, future studies could include labeled as opposed to unlabeled vignettes, which may change results as symptoms are clearly labeled as part of an illness. Overall, continuum beliefs may have different impacts on different levels of stigma (e.g., public stigma and self-stigma). It seems particularly relevant to assess the association between continuum beliefs and aspects of self-stigma, as self-stigma has been associated with medication adherence (37), depressive symptoms (38), and drinking refusal self-efficacy (39), among others, and thus is associated with prognosis.

### 4.3 Conclusion

The current study examines how the endorsement of continuum beliefs is related to illness recognition, treatment recommendations, and different aspects of stigma (e.g., stereotypes, emotions, and desired social distance). Predicted probabilities were used to depict effect sizes. Overall, increased continuum beliefs were strongly related to a decreased likelihood of desired social distance as well as decreased endorsement of various negative stereotypes. Besides associations between increased continuum beliefs and an increased likelihood for endorsing a lack of will power and more anger, we found associations with a reduced likelihood of illness recognition and treatment recommendations following the description of a person with symptoms of schizophrenia or depression. Considering the base levels and comparing direct effect sizes, the potentially beneficial and destigmatizing associations of continuum beliefs continue to be most important. Nevertheless, potentially harmful associations with a decreased likelihood of illness recognition and treatment recommendation should be considered in future observational and intervention studies.

## Data Availability

The raw data supporting the conclusions of this article will be made available by the authors, without undue reservation.
