# Supplementary material for: Differentiating the potential of continuum beliefs to reduce the public stigma of schizophrenia, depression, and alcohol dependence: associations with illness recognition and treatment recommendations
Source: Front Psychiatry. 2026 Sep 7;17:1869356. doi: 10.3389/fpsyt.2026.1869356 (PMC13590736; doi:10.3389/fpsyt.2026.1869356)
Supplement: Supplementary file 1 [file Table1.docx]

**Supplementary table 1**

Socio-demographic characteristics of the population sample.

|  | Total population 2010 ^a^ | Survey 2011  (n=3642) |
| --- | --- | --- |
| Gender |  |  |
| Men | 48.6 | 45.6 |
| Women | 51.4 | 54.4 |
|  |  |  |
| Age |  |  |
| 18-25 | 11.3 | 8.5 |
| 26-45 | 31.9 | 30.7 |
| 46-60 | 26.9 | 28.5 |
| >61 | 29.9 | 32.4 |
|  |  |  |
| Education ^b^ |  |  |
| Unknown/pupil | 1.0 | 0.7 |
| No schooling completed | 4.0 | 3.4 |
| 8/9 years of schooling | 38.5 | 38.9 |
| 10 years of schooling | 29.3 | 39.5 |
| 12/13 years of schooling | 27.1 | 17.5 |
|  |  |  |
| Marital status |  |  |
| Married | 51.9 | 53.8 |
| Divorced | 9.5 | 12.0 |
| Widowed | 9.1 | 11.9 |
| Single | 29.5 | 22.3 |
|  |  |  |

^a^ Data from the Statistical Office Germany

^b^ only persons >=20 years, population data for younger persons not available

**Supplementary tables 2 - 4**

Recognition of medical illness, recommendations for help-seeking and treatment, emotional reactions, stereotypes and desire for social distance after presentation of unlabelled case vignettes describing symptoms of **schizophrenia** (supplementary table 2), **depression** (supplementary table 3) or **alcohol dependence** (supplementary table 4) meeting DSM-IV criteria. Multinomial regression analyses, controlled for socio-demographic variables, probabilities of related categories with continuum belief at its mean (Base (%)) and after an increase of one standard deviation (Base + SD (%)), the resulting probability difference due to the effect of the continuum belief (Δ (%)) and its 95 % confidence interval (95% CI Δ). Total N in full models was 3551 – 3560 after missing values were excluded.

**Supplementary Table 2**

| Schizophrenia | | | | | | | | | | | | | | | | | | | | | | | | | |
| --- | --- | --- | --- | --- | --- | --- | --- | --- | --- | --- | --- | --- | --- | --- | --- | --- | --- | --- | --- | --- | --- | --- | --- | --- | --- |
|  |  |  | |  |  |  |  |  | |  |  |  |  |  | |  |  |  |  |  | |  |  |  | *Model fit* |
|  | | | Agree / Recommend | | |  | | | Neutral | | |  | | | Reject / Advise against | | |  | | | Don’t know | | |  |  |
|  | Base (%) | Base + SD (%) | | Δ (%) | 95% CI Δ |  | Base (%) | Base + SD (%) | | Δ (%) | 95% CI Δ |  | Base (%) | Base + SD (%) | | Δ (%) | 95% CI Δ |  | Base (%) | Base + SD (%) | | Δ (%) | 95% CI Δ |  | *Pseudo R²; p-value* |
| Medical illness | 83.7 | 79.7 | | -**4.0**** | -6.4 – 1.6 |  |  |  | |  |  |  | 7.2 | 10.2 | | **2.9**** | 1.1 – 4.8 |  | 9.0 | 10.1 | | 1.1 | -0.7 – 2.8 |  | *0.058; p<0.001* |
|  |  |  | |  |  |  |  |  | |  |  |  |  |  | |  |  |  |  |  | |  |  |  |  |
| *Help-seeking* |  |  | |  |  |  |  |  | |  |  |  |  |  | |  |  |  |  |  | |  |  |  |  |
| Psychiatrist | 82.0 | 75.6 | | **-6.4***** | -9.1 - -3 .8 |  | 9.6 | 13.7 | | **4.2***** | 2.0 – 6.3 |  | 5.9 | 8.4 | | **2.4**** | 0.7 – 4.1 |  | 2.5 | 2.3 | | -0.2 | -1.0 – 0.7 |  | *0.044; p<0.001* |
|  |  |  | |  |  |  |  |  | |  |  |  |  |  | |  |  |  |  |  | |  |  |  |  |
| *Treatment* |  |  | |  |  |  |  |  | |  |  |  |  |  | |  |  |  |  |  | |  |  |  |  |
| Psychotherapy | 82.1 | 80.5 | | -1.6 | -3.9 – 0.7 |  | 8.5 | 9.0 | | 0.5 | -1.1 – 2.2 |  | 4.3 | 5.3 | | 1.1 | -0.3 – 2.4 |  | 5.2 | 5.2 | | 0.0 | -1.2 – 1.3 |  | *0.023; p<0.001* |
| Psychotropic medication | 53.5 | 48.0 | | **-5.4***** | -8.3 – -2.6 |  | 21.1 | 22.9 | | 1.8 | -0.6 – 4.3 |  | 15.7 | 20.5 | | **4.8***** | 2.4 – 7.3 |  | 9.7 | 8.5 | | -1.2 | -2.7 – 0.3 |  | *0.036; p<0.001* |
|  |  |  | |  |  |  |  |  | |  |  |  |  |  | |  |  |  |  |  | |  |  |  |  |
| *Emotions* |  |  | |  |  |  |  |  | |  |  |  |  |  | |  |  |  |  |  | |  |  |  |  |
| He/she provokes fear | 37.4 | 35.2 | | -2.2 | -4.9 – 0.5 |  | 22.9 | 22.3 | | -0.6 | -2.9 – 1.8 |  | 39.7 | 42.5 | | 2.8 | -0.1 – 5.6 |  |  |  | |  |  |  | *0.025; p<0.001* |
| I feel uncomfortable | 49.0 | 42.9 | | **-6.0***** | -8.9 - -3.2 |  | 24.6 | 26.0 | | 1.4 | -1.2 – 3.9 |  | 26.5 | 31.1 | | **4.7**** | 1.9 – 7.4 |  |  |  | |  |  |  | *0.027; p<0.001* |
| I react angrily | 9.2 | 11.8 | | **2.5*** | 0.6 – 4.5 |  | 16.3 | 16.9 | | 0.6 | -1.6 – 2.7 |  | 74.5 | 71.4 | | **-3.1*** | -5.7 - -0.5 |  |  |  | |  |  |  | *0.041; p<0.001* |
| I feel annoyed by him/her | 13.0 | 12.4 | | -0.5 | -2.4 – 1.3 |  | 24.0 | 23.4 | | -0.6 | -2.9 – 1.8 |  | 63.1 | 64.1 | | 1.1 | -1.6 – 3.8 |  |  |  | |  |  |  | *0.026; p<0.001* |
| I feel sympathy for him/her | 22.3 | 33.2 | | **10.9***** | 7.9 – 13.9 |  | 38.9 | 38.6 | | -0.2 | -3.1 – 2.6 |  | 38.8 | 28.2 | | **-10.6***** | -13.2 - -8.1 |  |  |  | |  |  |  | *0.047; p<0.001* |
|  |  |  | |  |  |  |  |  | |  |  |  |  |  | |  |  |  |  |  | |  |  |  |  |
| Stereotypes |  |  | |  |  |  |  |  | |  |  |  |  |  | |  |  |  |  |  | |  |  |  |  |
| Dangerous | 22.6 | 21.3 | | -1.3 | -3.6 – 1.0 |  | 29.1 | 27.3 | | -1.8 | -4.3 – 0.7 |  | 48.3 | 51.5 | | **3.2*** | 0.3 – 6.0 |  |  |  | |  |  |  | *0.035; p<0.001* |
| Unpredictable | 48.7 | 42.0 | | **-6.7***** | -9.5 - -3.9 |  | 27.9 | 28.3 | | 0.4 | -2.2 – 3.0 |  | 23.3 | 29.7 | | **6.4***** | 3.6 – 9.1 |  |  |  | |  |  |  | *0.050; p<0.001* |
| Alien | 52.2 | 41.6 | | **-10.6***** | -13.5 - -7.7 |  | 24.8 | 30.0 | | **5.3***** | 2.5 – 8.0 |  | 23.1 | 28.4 | | **5.3***** | 2.6 – 8.0 |  |  |  | |  |  |  | *0.035; p<0.001* |
| Lack of will power | 30.9 | 36.0 | | **5.1***** | 2.2 – 7.9 |  | 25.9 | 25.9 | | 0.1 | -2.4 – 2.6 |  | 43.2 | 38.0 | | **-5.2***** | -7.9 - -2.4 |  |  |  | |  |  |  | *0.051; p<0.001* |
|  |  |  | |  |  |  |  |  | |  |  |  |  |  | |  |  |  |  |  | |  |  |  |  |
| *Social distance* |  |  | |  |  |  |  |  | |  |  |  |  |  | |  |  |  |  |  | |  |  |  |  |
| Introduce to a friend | 18.9 | 25.2 | | **6.3***** | 3.7 – 9.0 |  | 27.0 | 31.2 | | **4.2**** | 1.4 – 7.0 |  | 54.1 | 43.6 | | **-10.5***** | -13.4 - -7.6 |  |  |  | |  |  |  | *0.042; p<0.001* |

* p <0.05, ** p <0.01, *** p <0.001

**Supplementary Table 3**

|  | Depression | | | | | | | | | | | | | | | | | | |  |  |
| --- | --- | --- | --- | --- | --- | --- | --- | --- | --- | --- | --- | --- | --- | --- | --- | --- | --- | --- | --- | --- | --- |
|  |  |  |  |  |  |  |  |  |  |  |  |  |  |  |  |  |  |  |  |  |  |
|  | Agree / Recommend | | | |  | Neutral | | | |  | Reject / Advise against | | | |  | Don’t know | | | |  | *Model fit* |
|  | Base (%) | Base + SD (%) | Δ (%) | 95% CI Δ |  | Base (%) | Base + SD (%) | Δ (%) | 95% CI Δ |  | Base (%) | Base + SD (%) | Δ (%) | 95% CI Δ |  | Base (%) | Base + SD (%) | Δ (%) | 95% CI Δ |  | *Pseudo R²; p-value* |
| Medical illness | 65.1 | 62.5 | -2.6 | -5.4 – 0.3 |  |  |  |  |  |  | 21.2 | 24.4 | **3.2*** | 0.6 – 5.8 |  | 13.7 | 13.1 | -0.6 | -2.5 – 1.3 |  | *0.058; p<0.001* |
|  |  |  |  |  |  |  |  |  |  |  |  |  |  |  |  |  |  |  |  |  |  |
| *Help-seeking* |  |  |  |  |  |  |  |  |  |  |  |  |  |  |  |  |  |  |  |  |  |
| Psychiatrist | 67.3 | 65.2 | -2.1 | -4.9 – 0.7 |  | 15.3 | 13.9 | -1.4 | -3.3 – 0.5 |  | 14.2 | 17.4 | **3.2**** | 0.8 – 5.6 |  | 3.2 | 3.4 | 0.2 | -0.8 – 1.3 |  | *0.044; p<0.001* |
|  |  |  |  |  |  |  |  |  |  |  |  |  |  |  |  |  |  |  |  |  |  |
| *Treatment* |  |  |  |  |  |  |  |  |  |  |  |  |  |  |  |  |  |  |  |  |  |
| Psychotherapy | 71.0 | 67.7 | **-3.2*** | -6.0 - -0.4 |  | 12.3 | 11.9 | -0.4 | -2.2 – 1.5 |  | 10.8 | 12.4 | 1.5 | -0.5 – 3.6 |  | 5.9 | 8.0 | **2.1*** | 0.3 – 3.8 |  | *0.023; p<0.001* |
| Psychotropic medication | 35.5 | 32.2 | **-3.3*** | -5.9 - -0.7 |  | 21.5 | 22.1 | 0.6 | -1.8 – 3.0 |  | 31.9 | 34.9 | **3.0**** | 0.2 – 5.8 |  | 11.0 | 10.8 | -0.3 | -2.0 – 1.5 |  | *0.036; p<0.001* |
|  |  |  |  |  |  |  |  |  |  |  |  |  |  |  |  |  |  |  |  |  |  |
| *Emotions* |  |  |  |  |  |  |  |  |  |  |  |  |  |  |  |  |  |  |  |  |  |
| He/she provokes fear | 20.0 | 18.5 | -1.5 | -3.6 – 0.6 |  | 18.5 | 17.5 | -1.0 | -3.1 – 1.1 |  | 50.6 | 48.4 | -2.3 | -5.2 – 0.7 |  |  |  |  |  |  | *0.025; p<0.001* |
| I feel uncomfortable | 30.5 | 28.0 | **-2.5*** | -5.0 - -0.0 |  | 22.1 | 21.0 | -1.1 | -3.4 – 1.2 |  | 47.4 | 51.0 | **3.6*** | 0.8 – 6.5 |  |  |  |  |  |  | *0.027; p<0.001* |
| I react angrily | 9.0 | 9.8 | 0.8 | -1.0 – 2.5 |  | 12.8 | 12.7 | -0.1 | -2.0 – 1.8 |  | 78.2 | 77.5 | -0.7 | -3.1 – 1.8 |  |  |  |  |  |  | *0.041; p<0.001* |
| I feel annoyed by him/her | 9.1 | 10.9 | 1.7 | -0.2 – 3.6 |  | 18.7 | 18.5 | -0.2 | -2.4 – 2.0 |  | 72.1 | 70.6 | -1.5 | -4.1 – 1.2 |  |  |  |  |  |  | *0.026; p<0.001* |
| I feel sympathy for him/her | 31.1 | 40.7 | **9.6***** | 6.5 – 12.8 |  | 40.3 | 36.0 | **-4.3**** | -7.1 - - 1.6 |  | 28.6 | 23.3 | **-5.3***** | -7.6 - -3.0 |  |  |  |  |  |  | *0.047; p<0.001* |
|  |  |  |  |  |  |  |  |  |  |  |  |  |  |  |  |  |  |  |  |  |  |
| Stereotypes |  |  |  |  |  |  |  |  |  |  |  |  |  |  |  |  |  |  |  |  |  |
| Dangerous | 10.0 | 9.3 | -0.7 | -2.3 – 0.9 |  | 17.6 | 17.4 | -0.2 | -2.3 – 2.0 |  | 72.4 | 73.3 | 0.8 | -1.7 – 3.3 |  |  |  |  |  |  | *0.035; p<0.001* |
| Unpredictable | 21.8 | 21.1 | -0.7 | -3.0 – 1.6 |  | 25.8 | 24.3 | -1.5 | -3.9 – 0.9 |  | 52.5 | 54.6 | 2.2 | -0.7 – 5.0 |  |  |  |  |  |  | *0.050; p<0.001* |
| Alien | 26.8 | 23.9 | **-2.9*** | -5.2 - -0.6 |  | 29.2 | 31.1 | 1.9 | -0.9 – 4.6 |  | 43.9 | 45.0 | 1.1 | -1.8 – 3.9 |  |  |  |  |  |  | *0.035; p<0.001* |
| Lack of will power | 28.7 | 32.2 | **3.5*** | 0.7 – 6.3 |  | 25.7 | 25.4 | -0.3 | -2.8 – 2.2 |  | 45.6 | 42.4 | **-3.2*** | -6.0 - -0.4 |  |  |  |  |  |  | *0.051; p<0.001* |
|  |  |  |  |  |  |  |  |  |  |  |  |  |  |  |  |  |  |  |  |  |  |
| *Social distance* |  |  |  |  |  |  |  |  |  |  |  |  |  |  |  |  |  |  |  |  |  |
| Introduce to a friend | 31.1 | 36.9 | **5.8***** | 2.8 – 8.7 |  | 31.5 | 30.3 | -1.2 | -3.8 – 1.4 |  | 37.4 | 32.8 | -4.6** | -7.2 - -1.9 |  |  |  |  |  |  | *0.042; p<0.001* |

* p <0.05, ** p <0.01, *** p <0.001

**Supplementary Table 4**

|  | Alcohol dependence | | | | | | | | | | | | | | | | | | |  |  |
| --- | --- | --- | --- | --- | --- | --- | --- | --- | --- | --- | --- | --- | --- | --- | --- | --- | --- | --- | --- | --- | --- |
|  |  |  |  |  |  |  |  |  |  |  |  |  |  |  |  |  |  |  |  |  |  |
|  | Agree / Recommend | | | |  | Neutral | | | |  | Reject / Advise against | | | |  | Don’t know | | | |  | *Model fit* |
|  | Base (%) | Base + SD (%) | Δ (%) | 95% CI Δ |  | Base (%) | Base + SD (%) | Δ (%) | 95% CI Δ |  | Base (%) | Base + SD (%) | Δ (%) | 95% CI Δ |  | Base (%) | Base + SD (%) | Δ (%) | 95% CI Δ |  | *Pseudo R²; p-value* |
| Medical illness | 51.8 | 56.1 | **4.3**** | 1.4 – 7.2 |  |  |  |  |  |  | 32.1 | 28.7 | **-3.4**** | -6.0 - - 0.9 |  | 16.0 | 15.2 | -0.8 | -2.9 – 1.2 |  | *0.058; p<0.001* |
|  |  |  |  |  |  |  |  |  |  |  |  |  |  |  |  |  |  |  |  |  |  |
| *Help-seeking* |  |  |  |  |  |  |  |  |  |  |  |  |  |  |  |  |  |  |  |  |  |
| Psychiatrist | 52.5 | 53.2 | 0.7 | -2.2 – 3.6 |  | 21.7 | 22.8 | 1.1 | -1.4 – 3.6 |  | 20.7 | 19.0 | -1.6 | -3.8 – 0.6 |  | 5.2 | 5.0 | -0.2 | -1.4 – 1.0 |  | *0.044; p<0.001* |
|  |  |  |  |  |  |  |  |  |  |  |  |  |  |  |  |  |  |  |  |  |  |
| *Treatment* |  |  |  |  |  |  |  |  |  |  |  |  |  |  |  |  |  |  |  |  |  |
| Psychotherapy | 66.5 | 65.2 | -1.3 | -4.1 – 1.5 |  | 16.2 | 16.7 | 0.4 | -1.7 – 2.6 |  | 10.1 | 10.2 | 0.2 | -1.6 – 2.0 |  | 7.2 | 7.9 | 0.7 | -0.9 – 2.3 |  | *0.023; p<0.001* |
| Psychotropic medication | 28.4 | 31.9 | **3.5*** | 0.7 – 6.3 |  | 21.6 | 23.1 | 1.4 | -1.1 – 3.9 |  | 40.6 | 36.7 | **-3.9**** | -6.7 - -1.1 |  | 9.4 | 8.3 | -1.1 | -2.6 – 0.4 |  | *0.036; p<0.001* |
|  |  |  |  |  |  |  |  |  |  |  |  |  |  |  |  |  |  |  |  |  |  |
| *Emotions* |  |  |  |  |  |  |  |  |  |  |  |  |  |  |  |  |  |  |  |  |  |
| He/she provokes fear | 26.2 | 28.2 | 2.0 | -0.6 – 4.7 |  | 23.1 | 23.4 | 0.2 | -2.2 – 2.7 |  | 50.6 | 48.4 | -2.3 | -5.2 – 0.7 |  |  |  |  |  |  | *0.025; p<0.001* |
| I feel uncomfortable | 41.7 | 37.1 | **-4.7**** | -7.4 - -1.9 |  | 26.8 | 25.7 | -1.1 | -3.7 – 1.4 |  | 31.4 | 37.3 | **5.8***** | 2.9 – 8.7 |  |  |  |  |  |  | *0.027; p<0.001* |
| I react angrily | 24.2 | 23.7 | -0.6 | -3.0 – 1.9 |  | 22.5 | 19.7 | **-2.8*** | -5.0 - -0.6 |  | 53.2 | 56.6 | **3.4*** | 0.5 – 6.2 |  |  |  |  |  |  | *0.041; p<0.001* |
| I feel annoyed by him/her | 22.3 | 19.3 | **-3.0**** | -5.2 - -0.8 |  | 28.2 | 26.9 | -1.3 | -3.8 – 1.3 |  | 49.5 | 53.8 | **4.3**** | 1.4 – 7.2 |  |  |  |  |  |  | *0.026; p<0.001* |
| I feel sympathy for him/her | 16.7 | 24.7 | **8.0***** | 5.2 – 10.8 |  | 31.7 | 33.9 | 2.2 | -0.7 – 5.0 |  | 51.6 | 41.4 | **-10.2***** | -13.1 - -7.2 |  |  |  |  |  |  | *0.047; p<0.001* |
|  |  |  |  |  |  |  |  |  |  |  |  |  |  |  |  |  |  |  |  |  |  |
| Stereotypes |  |  |  |  |  |  |  |  |  |  |  |  |  |  |  |  |  |  |  |  |  |
| Dangerous | 23.7 | 23.9 | 0.2 | -2.3 – 2.7 |  | 29.6 | 31.1 | 1.5 | -1.2 – 4.2 |  | 46.7 | 45.0 | -1.7 | -4.6 – 1.2 |  |  |  |  |  |  | *0.035; p<0.001* |
| Unpredictable | 46.3 | 43.0 | **-3.2*** | -6.1 - -0.4 |  | 29.2 | 30.0 | 0.8 | -1.8 – 3.5 |  | 24.5 | 26.9 | 2.4 | -0.2 – 5.0 |  |  |  |  |  |  | *0.050; p<0.001* |
| Alien | 33.5 | 33.4 | -0.2 | -2.9 – 2.6 |  | 33.8 | 33.8 | -0.0 | -2.8 – 2.7 |  | 32.6 | 32.8 | 0.2 | -2.5 – 2.9 |  |  |  |  |  |  | *0.035; p<0.001* |
| Lack of will power | 59.9 | 60.0 | 0.1 | -2.7 – 3.0 |  | 22.0 | 23.6 | 1.5 | -1.0 - 4.0 |  | 18.1 | 16.5 | -1.6 | -3.7 – 0.4 |  |  |  |  |  |  | *0.051; p<0.001* |
|  |  |  |  |  |  |  |  |  |  |  |  |  |  |  |  |  |  |  |  |  |  |
| *Social distance* |  |  |  |  |  |  |  |  |  |  |  |  |  |  |  |  |  |  |  |  |  |
| Introduce to a friend | 15.1 | 20.7 | **5.6***** | 3.0 – 8.1 |  | 23.5 | 26.5 | **3.0*** | 0.4 – 5.7 |  | 61.4 | 52.8 | **-8.6***** | -11.6 - -5.6 |  |  |  |  |  |  | *0.042; p<0.001* |

* p <0.05, ** p <0.01, *** p <0.001
